# Down-regulation of pancreatic and duodenal homeobox-1 by somatostatin receptor subtype 5: a novel mechanism for inhibition of cellular proliferation and insulin secretion by somatostatin

**DOI:** 10.3389/fphys.2014.00226

**Published:** 2014-06-25

**Authors:** Guisheng Zhou, Jim Sinnett-Smith, Shi-He Liu, Juehua Yu, James Wu, Robbi Sanchez, Stephen J. Pandol, Ravinder Abrol, John Nemunaitis, Enrique Rozengurt, F. Charles Brunicardi

**Affiliations:** ^1^Division of General Surgery, Department of Surgery, David Geffen School of Medicine at University of CaliforniaLos Angeles, CA, USA; ^2^CURE: Digestive Disease Research Center, David Geffen School of Medicine at University of CaliforniaLos Angeles, CA, USA; ^3^Department of Medicine, David Geffen School of Medicine at University of CaliforniaLos Angeles, CA, USA; ^4^Department of Medicine at Cedars Sinai Medical CenterLos Angeles, CA, USA; ^5^Veterans AffairsLos Angeles, CA, USA; ^6^Materials and Process Simulation Center, California Institute of TechnologyPasadena, CA, USA; ^7^Gradalis, Inc.,Dallas, TX, USA; ^8^Mary Crowley Cancer Research CentersDallas, TX, USA

**Keywords:** G protein-coupled receptors, pancreatic and duodenal homeobox-1, single nucleotide polymorphisms, somatostatin, somatostatin receptor

## Abstract

Somatostatin (SST) is a regulatory peptide and acts as an endogenous inhibitory regulator of the secretory and proliferative responses of target cells. SST’s actions are mediated by a family of seven transmembrane domain G protein-coupled receptors that comprise five distinct subtypes (SSTR1-5). SSTR5 is one of the major SSTRs in the islets of Langerhans. Homeodomain-containing transcription factor pancreatic and duodenal homeobox-1 (PDX-1) is essential for pancreatic development, β cell differentiation, maintenance of normal β cell functions in adults and tumorigenesis. Recent studies show that SSTR5 acts as a negative regulator for PDX-1 expression and that SSTR5 mediates somatostatin’s inhibitory effect on cell proliferation and insulin expression/excretion through down-regulating PDX-1 expression. SSTR5 exerts its inhibitory effect on PDX-1 expression at both the transcriptional level by down-regulating PDX-1 mRNA and the post-translational level by enhancing PDX-1 ubiquitination. Identification of PDX-1 as a transcriptional target for SSTR5 may help in guiding the choice of therapeutic cancer treatments.

## Introduction

Somatostatin (SST) is a peptide hormone involved in a wide variety of biological functions including inhibition of endocrine and exocrine secretion; modulation of neurotransmission; motor and cognitive functions; inhibition of intestinal motility; absorption of nutrients and ions; vascular contractility; and regulation of cell proliferation, differentiation, inflammatory and immune responses (Patel, 1999; Wang et al., 2005a; Ballian et al., 2006, 2007; Florio, 2008). SSTs exert their actions through a group of five G protein-coupled transmembrane receptors, termed somatostatin receptor 1-5 (SSTR1-5) (Yamada et al., 1992; Yasuda et al., 1992; Raynor et al., 1993; Reisine and Bell, 1995; Moldovan et al., 1998). Following binding of SST, SSTRs undergo a cell type-specific conformational change, homo/heterodimerization (Rocheville et al., 2000; Pfeiffer et al., 2001; Duran-Prado et al., 2007), internalization (Peverelli et al., 2008), recruiting the G protein family members, and activation of downstream signaling pathways (Patel, 1999; Florio, 2008).

SSTR5 (Moldovan et al., 1998) is one of the major SSTRs in the islets of Langerhans. It is present in 87% of insulin-producing β-cells, 35% of glucagon-producing α-cells, and 75% of somatostatin-producing δ-cells and plays an essential role in mediating the inhibitory effect of somatostatin on insulin expression and secretion (Fagan et al., 1998). SSTR5 gene ablation results in marked increase in insulin secretion, causing basal hypoglycemia and circulating hyperinsulinemia (Wang et al., 2004, 2005a,b). Throughout the GI tract, SSTR5 has an anti-proliferative effect by inducing cell cycle arrest at the G1 phase via a mechanism that increases the production of retinoblastoma tumor suppressor protein and p21 (cyclin dependent kinase inhibitor) (Patel, 1999). SSTR5 knockout mice develop islet neoplasia associated with enlarged islets (Wang et al., 2004, 2005a,b). Due to its differential expression, SSTR5 is involved in tumorigenesis and drug responsiveness of a variety of human cancers including pancreatic cancer (Reubi et al., 1988; Li et al., 2011; Zhou et al., 2011a; Kaemmerer et al., 2013), pancreatic endocrine tumors (PETs) (Zhou et al., 2011b, 2012; Kaemmerer et al., 2013), pulmonary neuroendocrine tumors (Tsuta et al., 2012), gastroenteropancreatic neuroendocrine tumors (Kim et al., 2011a; Sclafani et al., 2011), small cell lung cancer (Oddstig et al., 2011), gallbladder cancer (Guo et al., 2013), colon cancer (Wang et al., 2013), endocrine pituitary tumors (Nishioka et al., 2011; Mayr et al., 2013; Chinezu et al., 2014), thyroid cancer (Ocak et al., 2013), corticotroph adenomas (Fleseriu and Petersenn, 2013), prostate cancer (Gu et al., 2010; Mazzucchelli et al., 2011; Lattanzio et al., 2013), and breast cancer (Gu et al., 2010). SSTR5 is also involved in the regulation of angiogenesis (Zatelli et al., 2001) and apoptosis (Qiu et al., 2006; Wang et al., 2013). This is consistent with microarray studies of pancreatic RNA from SSTR5^−/−^ mice in which ablation of SSTR5 results in up-regulation of proliferated and angiogenic genes and suppression of apoptotic genes (Patel et al., 2009).

Pancreatic and duodenal homeobox-1 (PDX-1) is an evolutionally conserved, homeodomain-containing β-cell-specific transcription factor (Leonard et al., 1993; Ohlsson et al., 1993; Olson et al., 1993; Miller et al., 1994; Marshak et al., 1996; Macfarlane et al., 1997). PDX-1 functions as a master regulator for a variety of cellular events including pancreatic development (Jonsson et al., 1994; Stoffers et al., 1997), β cell differentiation (Zhou et al., 2008) and postnatal β-cell function and survival (Ashizawa et al., 2004). PDX-1 expression in adults is essentially restricted to the nuclei of approximately 90% of insulin-producing islet β cells where it binds to the promoters of insulin (Ohlsson et al., 1993), glucose transporter 2 (Ohlsson et al., 1993), islet amyloid polypeptide (Serup et al., 1996; Macfarlane et al., 2000), and glucokinase (Watada et al., 1996). It regulates their expression, thus playing a critical role in maintaining mature β-cell function since all of these genes are critical for glucose sensing and insulin synthesis. As a result, PDX-1 plays an essential role in development of diabetes (Ahlgren et al., 1998; Brissova et al., 2002; Al-Quobaili and Montenarh, 2008; Gauthier et al., 2009). Increasingly, studies show that PDX-1 is also involved in tumorigenesis. PDX-1 is aberrantly overexpressed in a variety of human cancers including pancreatic, gastric, liver, colon, breast, prostate, kidney, lung, and ovarian cancer (Koizumi et al., 2003; Sakai et al., 2004; Wang et al., 2005c; Leys et al., 2006; Miyatsuka et al., 2006; Liu et al., 2007; Jonmarker et al., 2008) and pancreatic neuroendocrine tumor (Liu et al., 2012; Zhou et al., 2012). Moreover, PDX-1 overexpression in patients with cancers is significantly correlated with the pathological parameters (e.g., metastasis and histological grade) (Koizumi et al., 2003; Liu et al., 2007). In addition, PDX-1 is specifically increased in human placentas from intra-uterine growth restriction (IUGR) and preeclampsia (PE) + IUGR pregnancies (Buffat et al., 2007) and represses transcriptional activity of the T-box-containing transcription factor TBX15 in a methylation-dependent manner (Chelbi et al., 2011).

SST’s effects on cellular functions are at least in part mediated by its ability to regulate transcription factors. For example, octreotide, a somatostatin analog, inhibits mRNA expression of transcription factor c-fos and AP-1 binding activity stimulated by dibutyryl adenosine 3′,5′-cyclic monophosphate, serum and phorbol ester 12-*O*-tetradecanoylphorbol-13-acetate (TPA) (Todisco et al., 1994, 1995). In this mini review, we summarize recent findings that PDX-1 acts as another transcription factor that is subject to the inhibitory regulation by SSTR5-mediated somatostatin signaling.

## Negative regulation of PDX-1 by SSTR5

### SSTR5 down-regulates PDX-1

PDX-1 is subject to positive regulation by glucose (21), glucagon-like peptide 1 (GLP-1) (Buteau et al., 1999; Wang et al., 1999; Movassat et al., 2002; Li et al., 2005) and palmitic acid (Arantes et al., 2006). PDX-1 also is negatively regulated by DNA damage (Lebrun et al., 2005), oxidative stress (Boucher et al., 2006), and advanced glycation end-products (AGEs) (Puddu et al., 2010) under different physiological conditions. RPL-1980 is a SSTR5 specific agonist which proves that SSTR5 is the SSTR subtype responsible for inhibiting glucose-stimulated insulin secretion (Fagan et al., 1998; Zhou et al., 2011c). Recent studies show that RPL-1980 abolishes GLP-1-stimulated PDX-1 expression in mouse insulinoma β-TC6 cells (Zhou et al., 2012), indicating that SSTR5-mediated somatostatin signaling is a potential novel negative regulator for PDX-1 expression. Further biochemical and genetic studies confirm the negative regulation of PDX-1 expression by SSTR5 (Zhou et al., 2012). The evidence includes: (1) increased expression of SSTR5 inhibits PDX-1 expression likely due to increased PDX-1 ubiquitination; and (2) knockdown of SSTR5 by SSTR5 shRNA in β-TC6 cells results in increased PDX-1 expression and enhanced insulin secretion in response to a high concentration of glucose. These findings are consistent with the fact that SSTR5 mediates the inhibitory effect of somatostatin on insulin secretion (Fagan et al., 1998; Tirone et al., 2003). A SSTR5 knockdown-induced increase of PDX-1 expression in mouse insulinoma MIN6 cells is accompanied by elevated expression of cyclin E and cdk4 and decreased expression of p21, p27, and p53, supporting previous studies showing that SSTR5 inhibits cell proliferation (Fagan et al., 1998; Feanny et al., 2008) and promotes apoptosis (Qiu et al., 2006). This also is consistent with a previous study showing that SSTR5 contributes to the induction of cyclin-dependent kinase inhibitor p27 (Grant et al., 2008); and (3) genetic ablation of SSTR5 results in increased expression of PDX-1, which is accompanied by increased expression of insulin and proliferating cell nuclear antigen (PCNA) in *sstr5^−/−^* islets. Also consistent is the finding that all three SSTR5 knockout mice (general SSTR5*^−/−^*, β cell-specific SSTR5*^−/−^* and SSTR1/5*^−/−^*, SSTR1 and SSTR5 double knockout) develop islet hyperplasia, increased numbers of islets and islet neogenesis compared with littermate wild type controls (Wang et al., 2004, 2005a,b). Given the essential role of PDX-1 in insulin expression (Ashizawa et al., 2004; Kaneto et al., 2007) and cell proliferation (Feanny et al., 2008), these studies support a new concept that SSTR5 may mediate the inhibitory effect of somatostatin on insulin expression and secretion and cell proliferation through a mechanism involving inhibiting PDX-1 expression.

### SSTR5 P335l, a hypofunctional SNP, causes up-regulation of PDX-1

Single nucleotide polymorphisms (SNPs) are the most common type of genetic variations in the human genome, which can occur in all coding, non-coding and regulatory regions of a gene (Botstein and Risch, 2003). A number of SNPs of SSTR5 have been identified, including 20 missense variations (A19T, P34S, G37R, A40T, L48M, A52V, W105R, P109S, V180M, R229K, R234C, R248C, L251S, V267I, R312C, A327V, T333M, P335L, R339K and G357R) (http://www.ncbi.nlm.nih.gov/projects/SNP/snp_ref.cgi?chooseRs=allandgo=GoandlocusId=6755). Genome sequence analysis identified a number of SSTR5 gene mutations in human pancreatic cancer and pancreatic neuroendocrine tumors. Among them, SSTR5 P335L is a non-synonymous SNP resulting from a C to T change at the 1004th nucleotide of the human SSTR5 gene (Zhou et al., 2011c). The SNP widely exists in the human population and in patients with pancreatic cancer (Li et al., 2011; Zhou et al., 2011c) and pancreatic neuroendocrine tumors (Zhou et al., 2011b), which are race-dependent. SSTR5 P335L acts as a hypofunctional SNP since SSTR5 P335L enhances cell proliferation in contrast to wild-type SSTR5 (Zhou et al., 2011c). Moreover, SSTR5 P335L prevents the inhibitory effects of SSTR5 agonist RPL-1980 on cell proliferation of Mia PaCa-2 cells and glucose-stimulated insulin secretion from mouse insulinoma β-TC6 cells, while wild-type SSTR5 enhances the effects (Zhou et al., 2011c). Consistently, SSTR5 P335L enhances PDX-1 expression with an accompanied decreased PDX-1 ubiquitination (Zhou et al., 2012). These studies further confirm that SSTR5 is a negative regulator for PDX-1 expression.

### Down-regulation of PDX-1 by SSTR5 occurs at both transcriptional and post-translational levels

PDX-1 expression is controlled at epigenetic(Park et al., 2008; Ma et al., 2010; Yang et al., 2012), transcriptional (Wu et al., 1997; Gupta et al., 2008; Sun et al., 2008; da Silva Xavier et al., 2010), post-translational [phosphorylation (Lebrun et al., 2005; An et al., 2006, 2010; Boucher et al., 2006; Humphrey et al., 2010), ubiquitination (Lebrun et al., 2005; Boucher et al., 2006; Humphrey et al., 2010; Kim et al., 2011b), and sumoylation (Kishi et al., 2003)] levels. SSTR5 knockdown-induced increase of PDX-1 expression is accompanied by an increased expression of PDX-1 mRNA, while overexpression of SSTR5 inhibits PDX-1 mRNA expression (Zhou et al., 2012), indicating that SSTR5 acts as a negative regulator of PDX-1 at least partially through a mechanism of down-regulating PDX-1 mRNA. In addition, it is also found that wild-type SSTR5 enhances PDX-1 ubiquitiantion, while hypofunctional SSTR5 P335L SNP inhibits PDX-1 ubiquitination (Zhou et al., 2012). Thus, these studies demonstrate that SSTR5 acts as a negative regulator for PDX-1 expression and that SSTR5 exerts its inhibitory effect on PDX-1 expression at both transcriptional level (e.g., down-regulating PDX-1 mRNA) and post-translational level (e.g., enhancing PDX-1 ubiquitination and destabilizing PDX-1).

## Perspective

Identification of PDX-1 as a novel transcriptional target for SSTR5-mediated somatostatin signaling greatly enhances our understanding of the cellular actions of somatostatin. SSTR5 has been shown to regulate a specific set of genes linked to cell proliferation, apoptosis, angiogenesis, immunity and tumorigenesis (Patel et al., 2009). PDX-1 also regulates a group of genes related to cell proliferation, apoptosis, invasion and angiogenesis (Liu et al., 2011). Given the negative regulation of PDX-1 by SSTR5 (Zhou et al., 2012), it is likely that somatostatin exerts its cellular actions through regulating PDX-1-targeted genes with SSTR5. In addition to SSTR5, SSTs also exert their actions through SSTR1, 2, 3, and/or 4 under different cellular context. Therefore, it will be important to further determine if other SSTR-mediated cellular functions of somatostatin also involve PDX-1.

**Figure 1:**
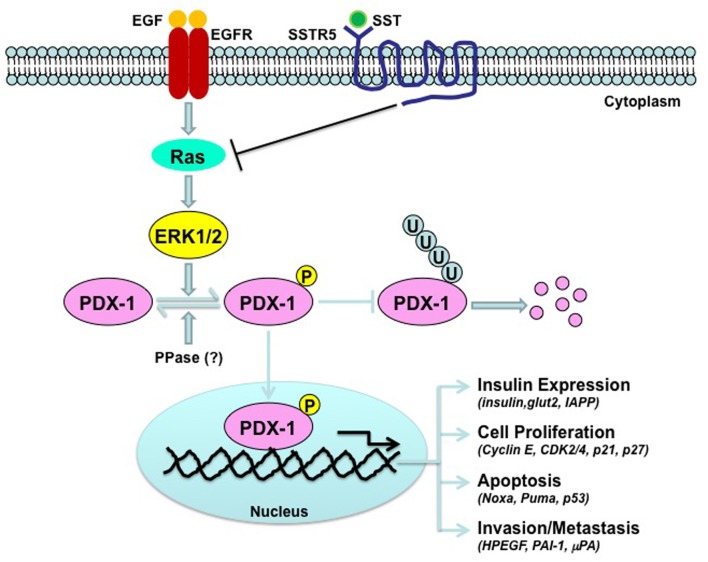
**Schematic depiction of the down-regulation of PDX-1 by SSTR5**. Epidermal growth factor (EGF) stimulates kinase activation of ERK MAP kinase, which, in turn, interacts with and phosphorylates PDX-1, leading to stabilization of PDX-1 by inhibiting PDX-1 ubiquitination. SSTR5-mediated somatostatin (SST) signaling inhibits EGF signaling by inhibiting small G protein Ras.

Little is known about the molecular mechanism by which SST/SSTR5 signaling down-regulates PDX-1. The observations that SSTR5 enhances, while SSTR5 P335L inhibits, PDX-1 ubiquitination (Zhou et al., 2012) suggest that regulation of PDX-1 ubiquitination is a key participant in the pathways that connects SSTR5 with PDX-1. It has been shown that PDX-1 ubiquitination is subject to regulation by phosphorylation. DNA-dependent protein kinase (DNA-PK) phosphorylates PDX-1 on Thr 11 and drives PDX-1 degradation by proteasome in response to DNA damage stimulation (Lebrun et al., 2005). Oxidative stress (H_2_O_2_)-stimulated, GSK-3-mediated phosphorylation of Ser 61 and Ser 66 also promotes PDX-1 proteasomal degradation (Boucher et al., 2006). ERK MAP kinases have kinase activity toward PDX-1 (Khoo et al., 2003). It has been shown that SST-mediated growth inhibition of human pituitary adenoma and GH3 cells is associated with the down-regulation of pERK and upregulation of p27 (Hubina et al., 2006). In addition, somatostatin exerts its anti-migratory and anti-invasive function through inhibition of ERK 1/2 signaling via the inhibition of small G protein Rac in SHSY5Y cells (Pola et al., 2003). Thus, ERK MAP kinase is one of downstream signaling pathways for somatostatin. It is, thus, reasonable to speculate that somatostatin/SSTR5 signaling may inhibit EGF-stimulated ERK kinase activation, leading to down-regulation of PDX-1 expression through a mechanism involved in the regulation of PDX-1 ubiquitination and stability (Figure 1). PDX-1 is a transcription factor for PDX-1 itself (Ashizawa et al., 2004). Thus, it is possible that down-regulation of PDX-1 mRNA by SSTR5 is due to the down-regulation of PDX-1 expression by SSTR5, which, in turn, results in decreased PDX-1 transcriptional activity. Alternatively, SSTR5 may directly down-regulate PDX-1 mRNA stability, leading to decreased PDX-1 mRNA expression.

SST is an endogenous antiproliferative agent and, thus, a promising antitumor agent (Florio, 2008; Pyronnet et al., 2008; Bodei et al., 2009; Modlin et al., 2009). SSTR5 and other SSTRs are widely and variably expressed in a variety of tumors such as gastroenteropancreatic tumors, pituitary tumors, and carcinoid tumors. Thus, these receptors represent the molecular basis for the clinical use of somatostatin analogs in the treatment of endocrine tumors. However, it has been challenging that only about 50% of patients with insulinoma are responsive to the treatment of somatostatin analog and lack long-term responsiveness. It could be due to the lack of expression of the target SSTRs on the tumor surface, since 10–50% of these tumors do not express SSTRs. Genetic variation(s) within the receptors that affect their cellular functions such as SSTR5 P335L, a hypofunctional SNP (Zhou et al., 2011c), may also contribute to the non-responsiveness to the treatment of somatostatin analogs. Silencing PDX-1 efficiently inhibits tumor cell proliferation *in vitro* and tumor growth *in vivo* (Liu et al., 2008, 2011), showing that PDX-1 is a promising therapeutic target in cancer treatment. Thus, it is reasonable to speculate that a combined SSTR5-based tumor therapeutic approach including both somatostatin analog and other inhibiting technology of PDX-1 [for example, RNA interference control of PDX-1 (Liu et al., 2008)] would help improve the traditional treatment with somatostatin analogs. In patients with wild-type SSTR5, knockdown PDX-1 by biPDX-1 shRNA would enhance the therapeutic effect of somatostatin analog. In the case that genetic variations exist such as SSTR5 P335L, efficient knockdown PDX-1 by shRNA would help bypass the hypofunctional effect of SSTR5 P335L. Thus, identification of PDX-1 as a transcriptional target for SSTR5 may help serve to guide the choice of therapeutic treatments.

### Conflict of interest statement

The authors declare that the research was conducted in the absence of any commercial or financial relationships that could be construed as a potential conflict of interest.
